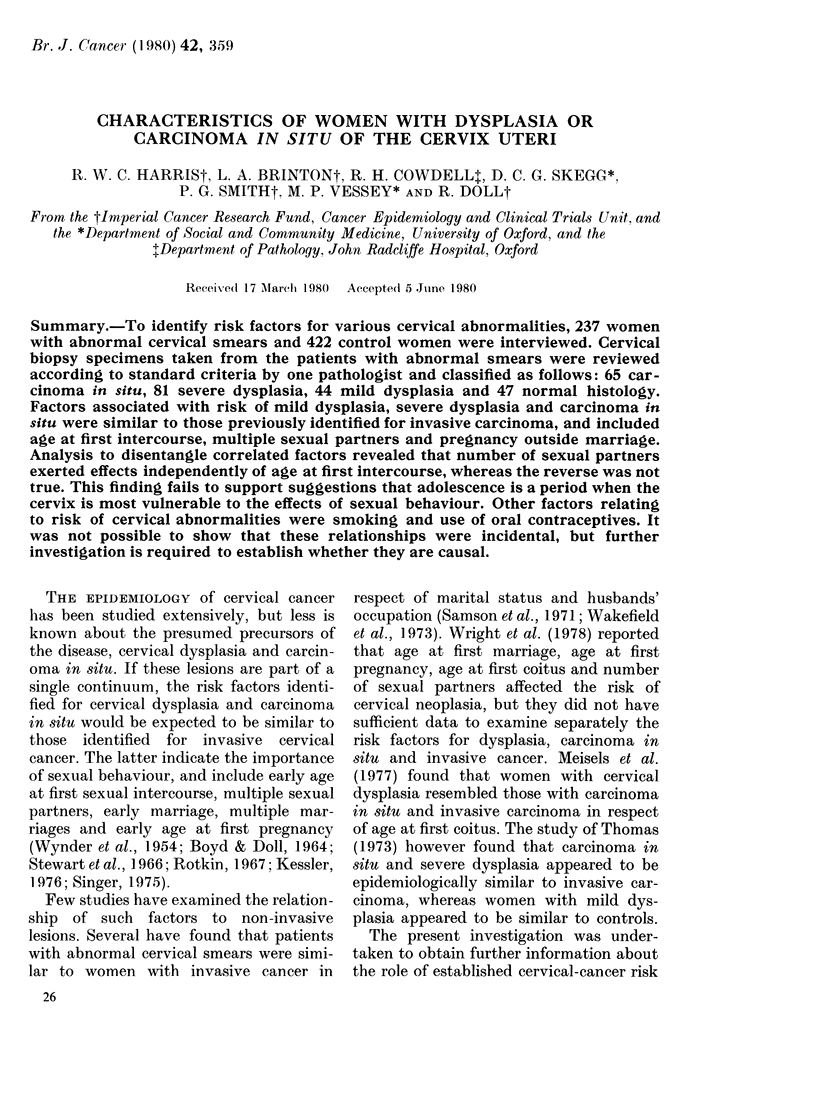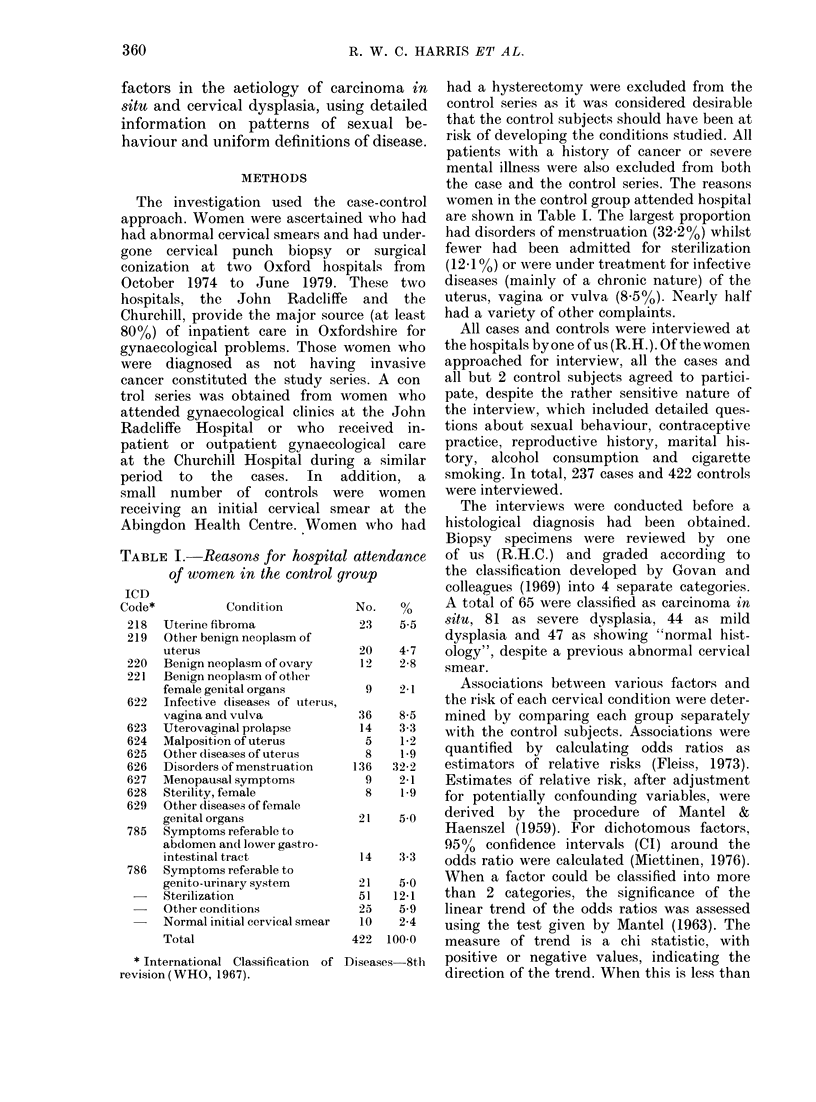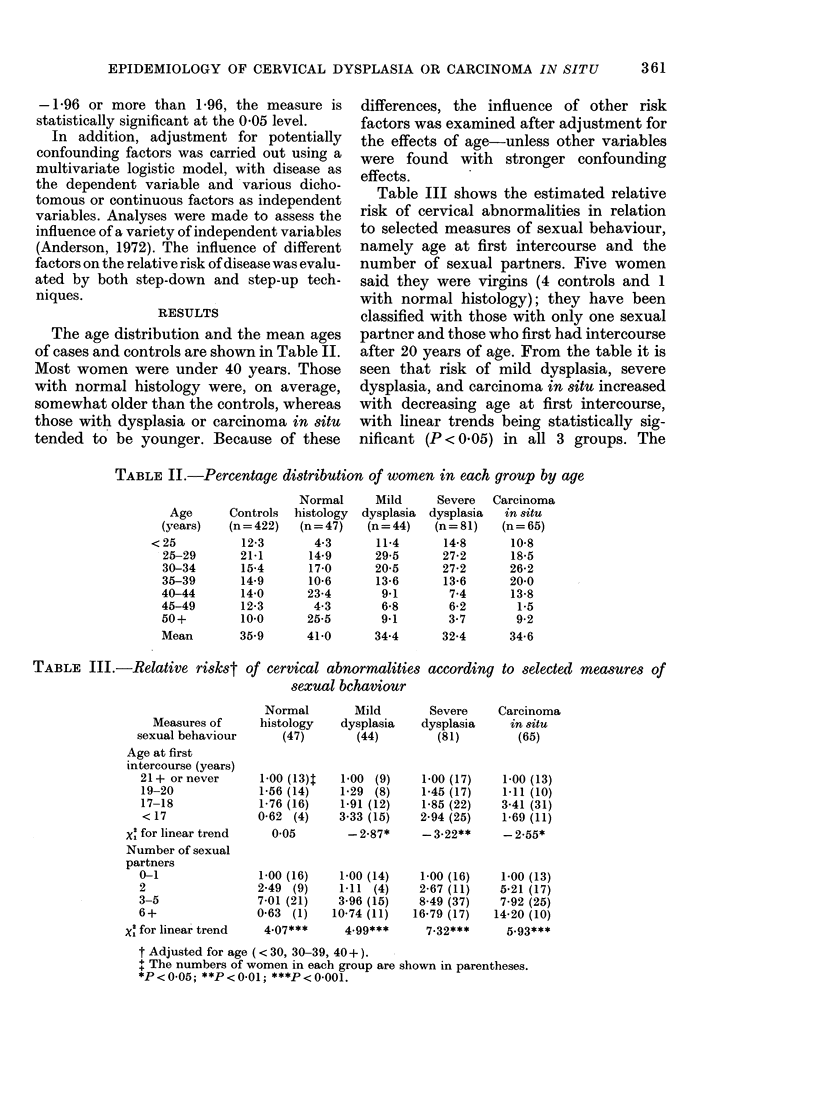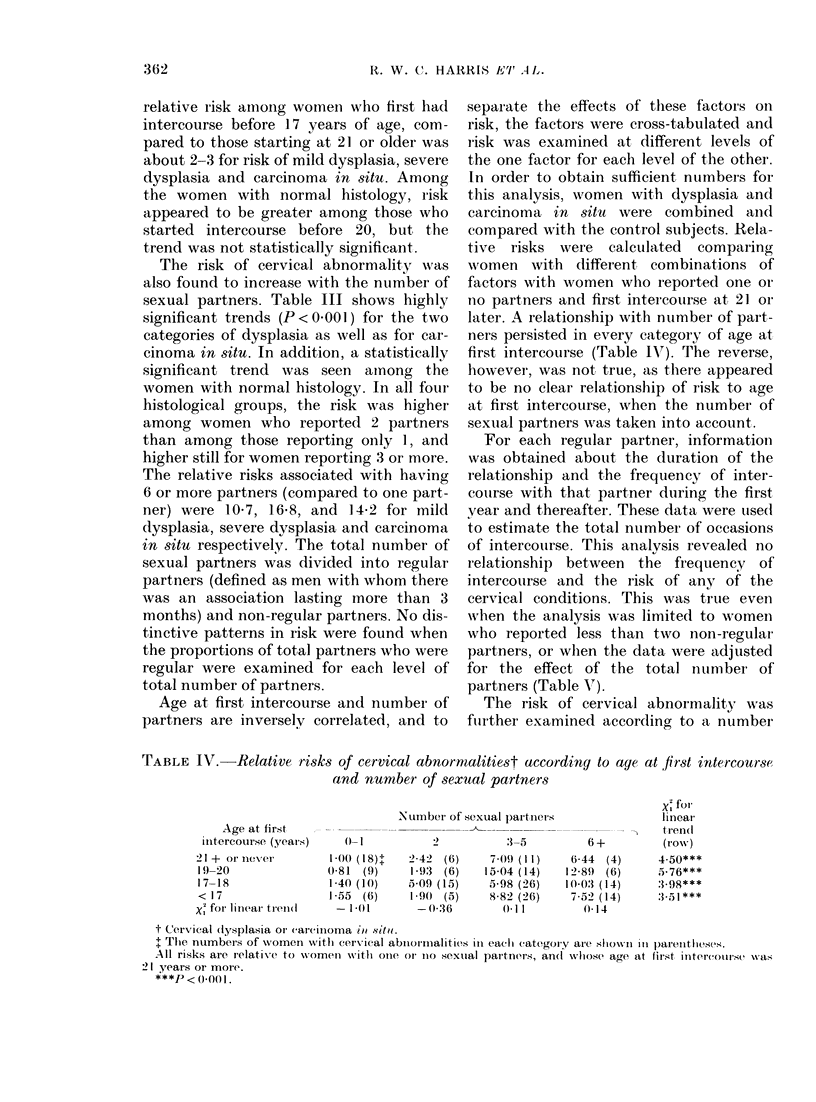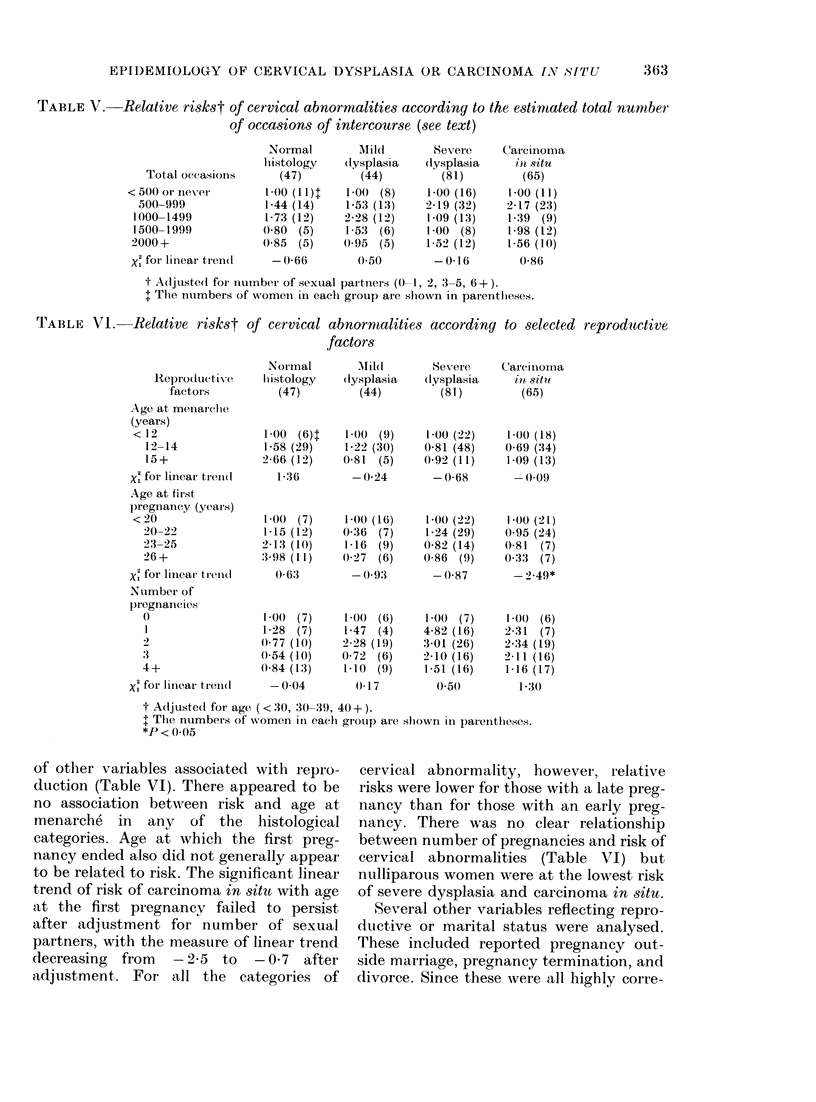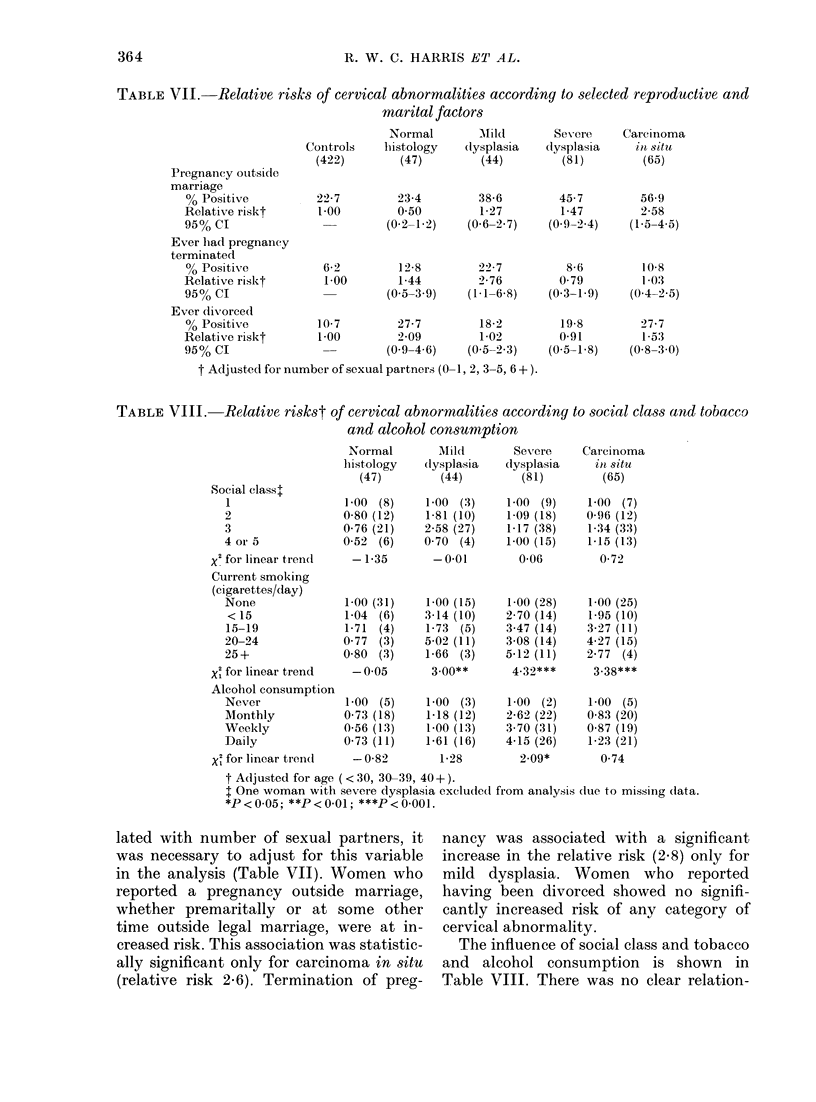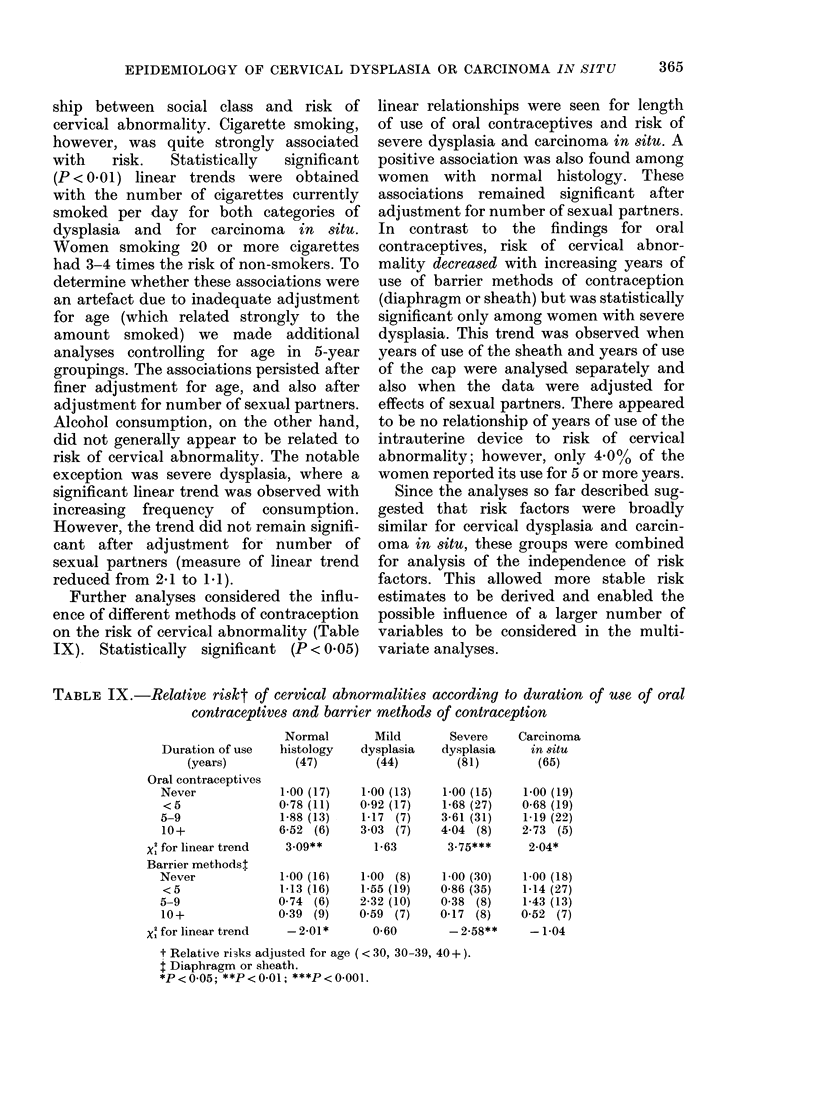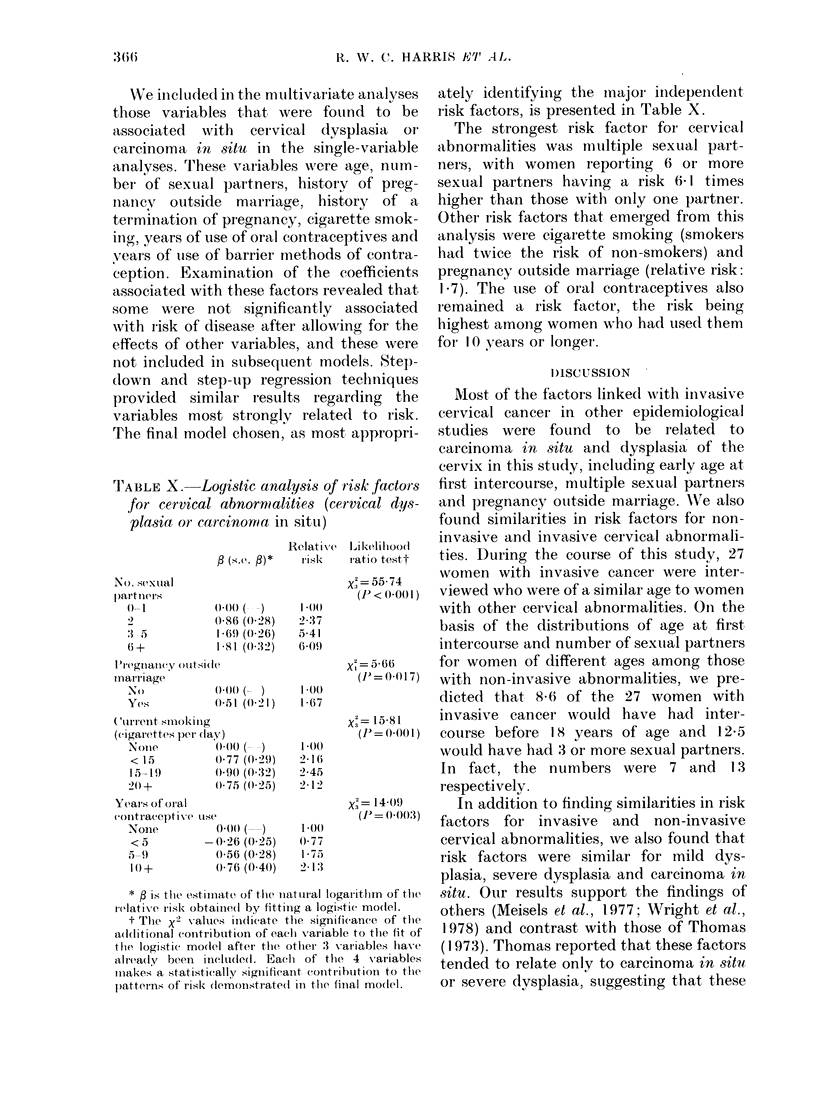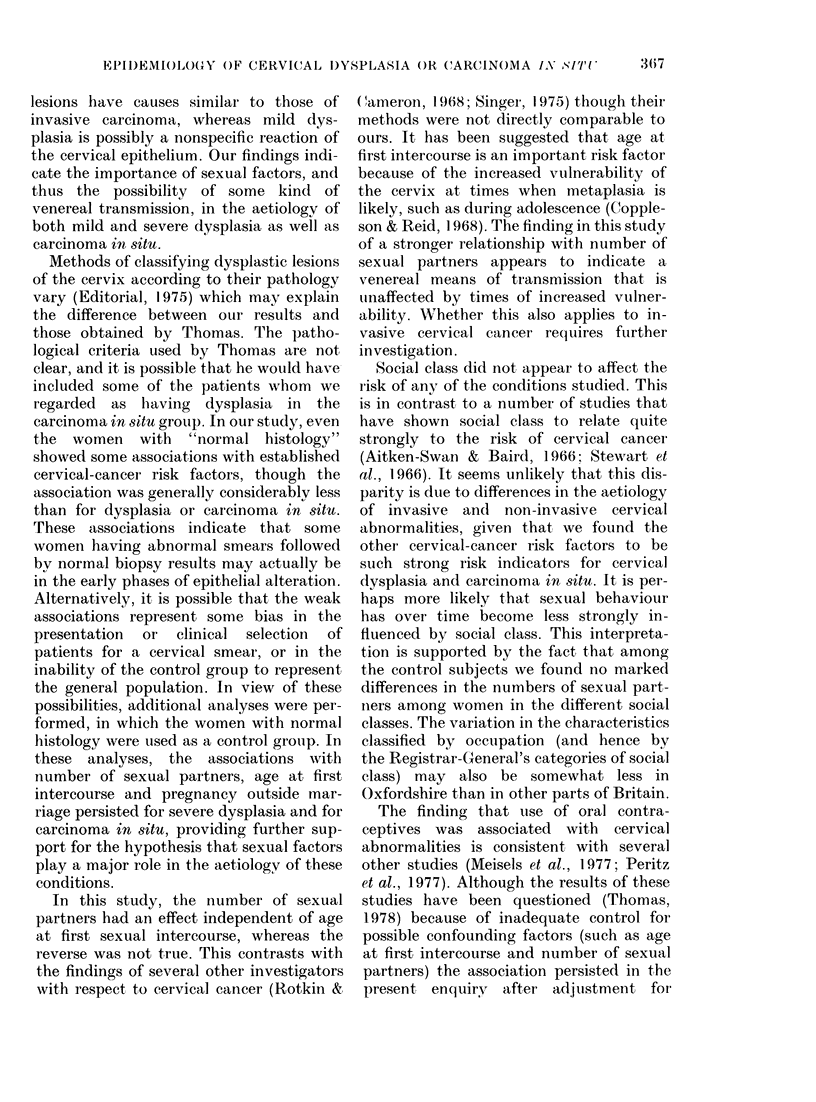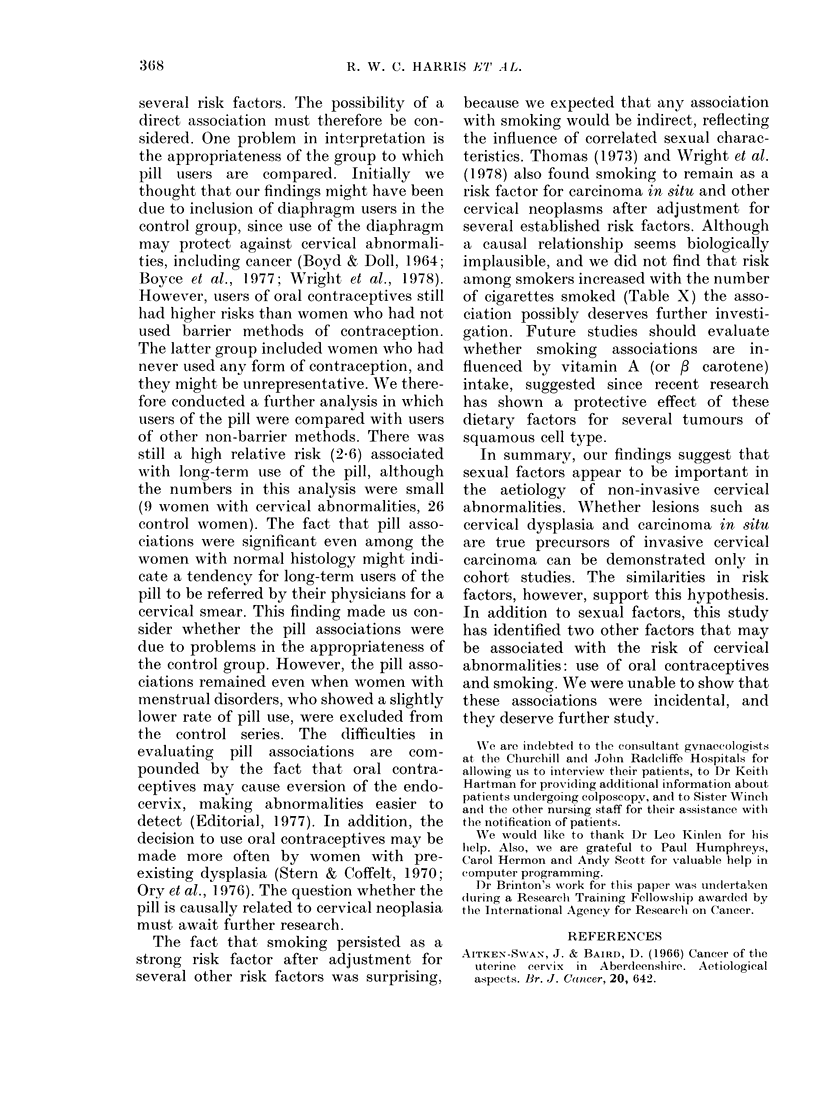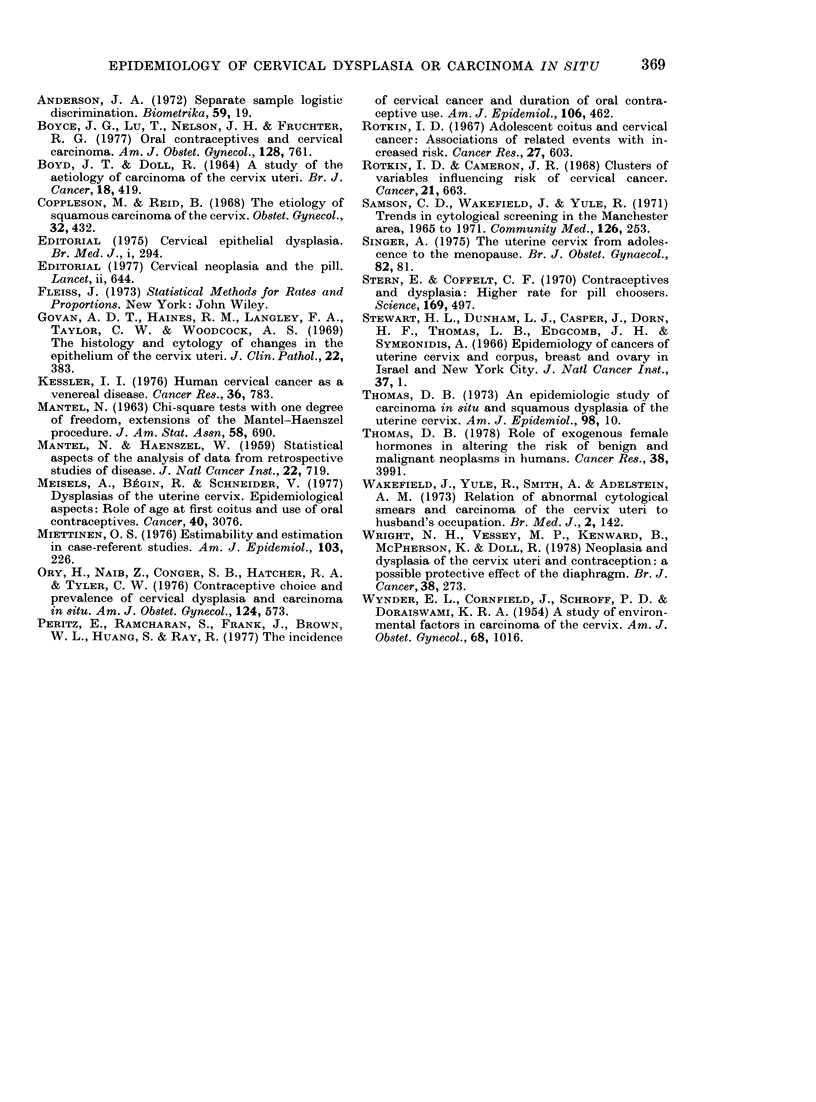# Characteristics of women with dysplasia or carcinoma in situ of the cervix uteri.

**DOI:** 10.1038/bjc.1980.246

**Published:** 1980-09

**Authors:** R. W. Harris, L. A. Brinton, R. H. Cowdell, D. C. Skegg, P. G. Smith, M. P. Vessey, R. Doll

## Abstract

To identify risk factors for various cervical abnormalities, 237 women with abnoromal cervical smears and 422 control women were interviewed. Cervical biopsy specimens taken from the patients with abnormal smears were reviewed according to standard criteria by one pathologist and classified as follows: 65 carcinoma in situ, 81 severe dysplasia, 44 mild dysplasia and 47 normal histology. Factors associated with risk of mild dysplasia, severe dysplasia and carcinoma in situ were similar to those previously identified for invasive carcinoma, and included age at first intercourse, multiple sexual partners and pregnancy outside marriage. Analysis to disentangle correlated factors revealed that number of sexual partners exerted effects independently of age at first intercourse, whereas the reverse was not true. This finding fails to support suggestions that adolescence is a period when the cervix is most vulnerable to the effects of sexual behaviour. Other factors relating to risk of cervical abnormalities were smoking and use of oral contraceptives. It was not possible to show that these relationships were incidental, but further investigation is required to establish whether they are causal.


*Br. J. Cancer* (1980) **42**, 359

### CHARACTERISTICS OF WOMEN WITH DYSPLASIA OR CARCINOMA *IN SITU* OF THE CERVIX UTERI

R. W. C. HARRIS†, L. A. BRINTON†, R. H. COWDELL‡, D. C. G. SKEGG*, P. G. SMITH†, M. P. VESSEY* AND R. DOLL†

*From the †Imperial Cancer Research Fund, Cancer Epidemiology and Clinical Trials Unit, and the *Department of Social and Community Medicine, University of Oxford, and the ‡Department of Pathology, John Radcliffe Hospital, Oxford*

Received 17 March 1980 Accepted 5 June 1980

**Summary.—To identify risk factors for various cervical abnormalities, 237 women with abnormal cervical smears and 422 control women were interviewed. Cervical biopsy specimens taken from the patients with abnormal smears were reviewed according to standard criteria by one pathologist and classified as follows: 65 carcinoma *in situ*, 81 severe dysplasia, 44 mild dysplasia and 47 normal histology. Factors associated with risk of mild dysplasia, severe dysplasia and carcinoma *in situ* were similar to those previously identified for invasive carcinoma, and included age at first intercourse, multiple sexual partners and pregnancy outside marriage. Analysis to disentangle correlated factors revealed that number of sexual partners exerted effects independently of age at first intercourse, whereas the reverse was not true. This finding fails to support suggestions that adolescence is a period when the cervix is most vulnerable to the effects of sexual behaviour. Other factors relating to risk of cervical abnormalities were smoking and use of oral contraceptives. It was not possible to show that these relationships were incidental, but further investigation is required to establish whether they are causal.**

THE EPIDEMIOLOGY of cervical cancer has been studied extensively, but less is known about the presumed precursors of the disease, cervical dysplasia and carcinoma *in situ*. If these lesions are part of a single continuum, the risk factors identified for cervical dysplasia and carcinoma *in situ* would be expected to be similar to those identified for invasive cervical cancer. The latter indicate the importance of sexual behaviour, and include early age at first sexual intercourse, multiple sexual partners, early marriage, multiple marriages and early age at first pregnancy (Wynder *et al.*, 1954; Boyd & Doll, 1964; Stewart *et al.*, 1966; Rotkin, 1967; Kessler, 1976; Singer, 1975).

Few studies have examined the relationship of such factors to non-invasive lesions. Several have found that patients with abnormal cervical smears were similar to women with invasive cancer in respect of marital status and husbands' occupation (Samson *et al.*, 1971; Wakefield *et al.*, 1973). Wright *et al.* (1978) reported that age at first marriage, age at first pregnancy, age at first coitus and number of sexual partners affected the risk of cervical neoplasia, but they did not have sufficient data to examine separately the risk factors for dysplasia, carcinoma *in situ* and invasive cancer. Meisels *et al.* (1977) found that women with cervical dysplasia resembled those with carcinoma *in situ* and invasive carcinoma in respect of age at first coitus. The study of Thomas (1973) however found that carcinoma *in situ* and severe dysplasia appeared to be epidemiologically similar to invasive carcinoma, whereas women with mild dysplasia appeared to be similar to controls.

The present investigation was undertaken to obtain further information about the role of established cervical-cancer risk factors in the aetiology of carcinoma *in situ* and cervical dysplasia, using detailed information on patterns of sexual behaviour and uniform definitions of disease.

### METHODS

The investigation used the case-control approach. Women were ascertained who had had abnormal cervical smears and had undergone cervical punch biopsy or surgical conization at two Oxford hospitals from October 1974 to June 1979. These two hospitals, the John Radcliffe and the Churchill, provide the major source (at least 80%) of inpatient care in Oxfordshire for gynaecological problems. Those women who were diagnosed as not having invasive cancer constituted the study series. A control series was obtained from women who attended gynaecological clinics at the John Radcliffe Hospital or who received inpatient or outpatient gynaecological care at the Churchill Hospital during a similar period to the cases. In addition, a small number of controls were women receiving an initial cervical smear at the Abingdon Health Centre. Women who had had a hysterectomy were excluded from the control series as it was considered desirable that the control subjects should have been at risk of developing the conditions studied. All patients with a history of cancer or severe mental illness were also excluded from both the case and the control series. The reasons women in the control group attended hospital are shown in Table I. The largest proportion had disorders of menstruation (32·2%) whilst fewer had been admitted for sterilization (12·1%) or were under treatment for infective diseases (mainly of a chronic nature) of the uterus, vagina or vulva (8·5%). Nearly half had a variety of other complaints.

TABLE I.—*Reasons for hospital attendance of women in the control group*

| ICD Code* | Condition | No. | % |
|---|---|---|---|
| 218 | Uterine fibroma | 23 | 5·5 |
| 219 | Other benign neoplasm of uterus | 20 | 4·7 |
| 220 | Benign neoplasm of ovary | 12 | 2·8 |
| 221 | Benign neoplasm of other female genital organs | 9 | 2·1 |
| 622 | Infective diseases of uterus, vagina and vulva | 36 | 8·5 |
| 623 | Uterovaginal prolapse | 14 | 3·3 |
| 624 | Malposition of uterus | 5 | 1·2 |
| 625 | Other diseases of uterus | 8 | 1·9 |
| 626 | Disorders of menstruation | 136 | 32·2 |
| 627 | Menopausal symptoms | 9 | 2·1 |
| 628 | Sterility, female | 8 | 1·9 |
| 629 | Other diseases of female genital organs | 21 | 5·0 |
| 785 | Symptoms referable to abdomen and lower gastro-intestinal tract | 14 | 3·3 |
| 786 | Symptoms referable to genito-urinary system | 21 | 5·0 |
| — | Sterilization | 51 | 12·1 |
| — | Other conditions | 25 | 5·9 |
| — | Normal initial cervical smear | 10 | 2·4 |
| | Total | 422 | 100·0 |

* International Classification of Diseases—8th revision (WHO, 1967).

All cases and controls were interviewed at the hospitals by one of us (R.H.). Of the women approached for interview, all the cases and all but 2 control subjects agreed to participate, despite the rather sensitive nature of the interview, which included detailed questions about sexual behaviour, contraceptive practice, reproductive history, marital history, alcohol consumption and cigarette smoking. In total, 237 cases and 422 controls were interviewed.

The interviews were conducted before a histological diagnosis had been obtained. Biopsy specimens were reviewed by one of us (R.H.C.) and graded according to the classification developed by Govan and colleagues (1969) into 4 separate categories. A total of 65 were classified as carcinoma *in situ*, 81 as severe dysplasia, 44 as mild dysplasia and 47 as showing "normal histology", despite a previous abnormal cervical smear.

Associations between various factors and the risk of each cervical condition were determined by comparing each group separately with the control subjects. Associations were quantified by calculating odds ratios as estimators of relative risks (Fleiss, 1973). Estimates of relative risk, after adjustment for potentially confounding variables, were derived by the procedure of Mantel & Haenszel (1959). For dichotomous factors, 95% confidence intervals (CI) around the odds ratio were calculated (Miettinen, 1976). When a factor could be classified into more than 2 categories, the significance of the linear trend of the odds ratios was assessed using the test given by Mantel (1963). The measure of trend is a chi statistic, with positive or negative values, indicating the direction of the trend. When this is less than

−1·96 or more than 1·96, the measure is statistically significant at the 0·05 level.

In addition, adjustment for potentially confounding factors was carried out using a multivariate logistic model, with disease as the dependent variable and various dichotomous or continuous factors as independent variables. Analyses were made to assess the influence of a variety of independent variables (Anderson, 1972). The influence of different factors on the relative risk of disease was evaluated by both step-down and step-up techniques.

### RESULTS

The age distribution and the mean ages of cases and controls are shown in Table II. Most women were under 40 years. Those with normal histology were, on average, somewhat older than the controls, whereas those with dysplasia or carcinoma *in situ* tended to be younger. Because of these differences, the influence of other risk factors was examined after adjustment for the effects of age—unless other variables were found with stronger confounding effects.

Table III shows the estimated relative risk of cervical abnormalities in relation to selected measures of sexual behaviour, namely age at first intercourse and the number of sexual partners. Five women said they were virgins (4 controls and 1 with normal histology); they have been classified with those with only one sexual partner and those who first had intercourse after 20 years of age. From the table it is seen that risk of mild dysplasia, severe dysplasia, and carcinoma *in situ* increased with decreasing age at first intercourse, with linear trends being statistically significant ($P < 0{\cdot}05$) in all 3 groups. The

TABLE II.—*Percentage distribution of women in each group by age*

| Age (years) | Controls (n = 422) | Normal histology (n = 47) | Mild dysplasia (n = 44) | Severe dysplasia (n = 81) | Carcinoma *in situ* (n = 65) |
|---|---|---|---|---|---|
| < 25 | 12·3 | 4·3 | 11·4 | 14·8 | 10·8 |
| 25–29 | 21·1 | 14·9 | 29·5 | 27·2 | 18·5 |
| 30–34 | 15·4 | 17·0 | 20·5 | 27·2 | 26·2 |
| 35–39 | 14·9 | 10·6 | 13·6 | 13·6 | 20·0 |
| 40–44 | 14·0 | 23·4 | 9·1 | 7·4 | 13·8 |
| 45–49 | 12·3 | 4·3 | 6·8 | 6·2 | 1·5 |
| 50+ | 10·0 | 25·5 | 9·1 | 3·7 | 9·2 |
| Mean | 35·9 | 41·0 | 34·4 | 32·4 | 34·6 |

TABLE III.—*Relative risks† of cervical abnormalities according to selected measures of sexual bchaviour*

| Measures of sexual behaviour | Normal histology (47) | Mild dysplasia (44) | Severe dysplasia (81) | Carcinoma *in situ* (65) |
|---|---|---|---|---|
| Age at first intercourse (years) | | | | |
| 21+ or never | 1·00 (13)‡ | 1·00 (9) | 1·00 (17) | 1·00 (13) |
| 19–20 | 1·56 (14) | 1·29 (8) | 1·45 (17) | 1·11 (10) |
| 17–18 | 1·76 (16) | 1·91 (12) | 1·85 (22) | 3·41 (31) |
| < 17 | 0·62 (4) | 3·33 (15) | 2·94 (25) | 1·69 (11) |
| $\chi^2_1$ for linear trend | 0·05 | −2·87* | −3·22** | −2·55* |
| Number of sexual partners | | | | |
| 0–1 | 1·00 (16) | 1·00 (14) | 1·00 (16) | 1·00 (13) |
| 2 | 2·49 (9) | 1·11 (4) | 2·67 (11) | 5·21 (17) |
| 3–5 | 7·01 (21) | 3·96 (15) | 8·49 (37) | 7·92 (25) |
| 6+ | 0·63 (1) | 10·74 (11) | 16·79 (17) | 14·20 (10) |
| $\chi^2_1$ for linear trend | 4·07*** | 4·99*** | 7·32*** | 5·93*** |

† Adjusted for age (< 30, 30–39, 40+).
‡ The numbers of women in each group are shown in parentheses.
*$P < 0{\cdot}05$; **$P < 0{\cdot}01$; ***$P < 0{\cdot}001$.

relative risk among women who first had intercourse before 17 years of age, compared to those starting at 21 or older was about 2–3 for risk of mild dysplasia, severe dysplasia and carcinoma *in situ*. Among the women with normal histology, risk appeared to be greater among those who started intercourse before 20, but the trend was not statistically significant.

The risk of cervical abnormality was also found to increase with the number of sexual partners. Table III shows highly significant trends ($P < 0{\cdot}001$) for the two categories of dysplasia as well as for carcinoma *in situ*. In addition, a statistically significant trend was seen among the women with normal histology. In all four histological groups, the risk was higher among women who reported 2 partners than among those reporting only 1, and higher still for women reporting 3 or more. The relative risks associated with having 6 or more partners (compared to one partner) were 10·7, 16·8, and 14·2 for mild dysplasia, severe dysplasia and carcinoma *in situ* respectively. The total number of sexual partners was divided into regular partners (defined as men with whom there was an association lasting more than 3 months) and non-regular partners. No distinctive patterns in risk were found when the proportions of total partners who were regular were examined for each level of total number of partners.

Age at first intercourse and number of partners are inversely correlated, and to separate the effects of these factors on risk, the factors were cross-tabulated and risk was examined at different levels of the one factor for each level of the other. In order to obtain sufficient numbers for this analysis, women with dysplasia and carcinoma *in situ* were combined and compared with the control subjects. Relative risks were calculated comparing women with different combinations of factors with women who reported one or no partners and first intercourse at 21 or later. A relationship with number of partners persisted in every category of age at first intercourse (Table IV). The reverse, however, was not true, as there appeared to be no clear relationship of risk to age at first intercourse, when the number of sexual partners was taken into account.

For each regular partner, information was obtained about the duration of the relationship and the frequency of intercourse with that partner during the first year and thereafter. These data were used to estimate the total number of occasions of intercourse. This analysis revealed no relationship between the frequency of intercourse and the risk of any of the cervical conditions. This was true even when the analysis was limited to women who reported less than two non-regular partners, or when the data were adjusted for the effect of the total number of partners (Table V).

The risk of cervical abnormality was further examined according to a number

TABLE IV.—*Relative risks of cervical abnormalities† according to age at first intercourse and number of sexual partners*

| Age at first intercourse (years) | Number of sexual partners 0–1 | 2 | 3–5 | 6+ | $\chi^2_1$ for linear trend (row) |
|---|---|---|---|---|---|
| 21+ or never | 1·00 (18)‡ | 2·42 (6) | 7·09 (11) | 6·44 (4) | 4·50*** |
| 19–20 | 0·81 (9) | 1·93 (6) | 15·04 (14) | 12·89 (6) | 5·76*** |
| 17–18 | 1·40 (10) | 5·09 (15) | 5·98 (26) | 10·03 (14) | 3·98*** |
| < 17 | 1·55 (6) | 1·90 (5) | 8·82 (26) | 7·52 (14) | 3·51*** |
| $\chi^2_1$ for linear trend | −1·01 | −0·36 | 0·11 | 0·14 | |

† Cervical dysplasia or carcinoma *in situ*.
‡ The numbers of women with cervical abnormalities in each category are shown in parentheses.
All risks are relative to women with one or no sexual partners, and whose age at first intercourse was 21 years or more.
***$P < 0{\cdot}001$.

TABLE V.—*Relative risks*† *of cervical abnormalities according to the estimated total number of occasions of intercourse (see text)*

| Total occasions | Normal histology (47) | Mild dysplasia (44) | Severe dysplasia (81) | Carcinoma *in situ* (65) |
|---|---|---|---|---|
| < 500 or never | 1·00 (11)‡ | 1·00 (8) | 1·00 (16) | 1·00 (11) |
| 500–999 | 1·44 (14) | 1·53 (13) | 2·19 (32) | 2·17 (23) |
| 1000–1499 | 1·73 (12) | 2·28 (12) | 1·09 (13) | 1·39 (9) |
| 1500–1999 | 0·80 (5) | 1·53 (6) | 1·00 (8) | 1·98 (12) |
| 2000+ | 0·85 (5) | 0·95 (5) | 1·52 (12) | 1·56 (10) |
| $\chi_1^2$ for linear trend | −0·66 | 0·50 | −0·16 | 0·86 |

† Adjusted for number of sexual partners (0–1, 2, 3–5, 6+).
‡ The numbers of women in each group are shown in parentheses.

TABLE VI.—*Relative risks*† *of cervical abnormalities according to selected reproductive factors*

| Reproductive factors | Normal histology (47) | Mild dysplasia (44) | Severe dysplasia (81) | Carcinoma *in situ* (65) |
|---|---|---|---|---|
| Age at menarche (years) | | | | |
| < 12 | 1·00 (6)‡ | 1·00 (9) | 1·00 (22) | 1·00 (18) |
| 12–14 | 1·58 (29) | 1·22 (30) | 0·81 (48) | 0·69 (34) |
| 15+ | 2·66 (12) | 0·81 (5) | 0·92 (11) | 1·09 (13) |
| $\chi_1^2$ for linear trend | 1·36 | −0·24 | −0·68 | −0·09 |
| Age at first pregnancy (years) | | | | |
| < 20 | 1·00 (7) | 1·00 (16) | 1·00 (22) | 1·00 (21) |
| 20–22 | 1·15 (12) | 0·36 (7) | 1·24 (29) | 0·95 (24) |
| 23–25 | 2·13 (10) | 1·16 (9) | 0·82 (14) | 0·81 (7) |
| 26+ | 3·98 (11) | 0·27 (6) | 0·86 (9) | 0·33 (7) |
| $\chi_1^2$ for linear trend | 0·63 | −0·93 | −0·87 | −2·49* |
| Number of pregnancies | | | | |
| 0 | 1·00 (7) | 1·00 (6) | 1·00 (7) | 1·00 (6) |
| 1 | 1·28 (7) | 1·47 (4) | 4·82 (16) | 2·31 (7) |
| 2 | 0·77 (10) | 2·28 (19) | 3·01 (26) | 2·34 (19) |
| 3 | 0·54 (10) | 0·72 (6) | 2·10 (16) | 2·11 (16) |
| 4+ | 0·84 (13) | 1·10 (9) | 1·51 (16) | 1·16 (17) |
| $\chi_1^2$ for linear trend | −0·04 | 0·17 | 0·50 | 1·30 |

† Adjusted for age (< 30, 30–39, 40+).
‡ The numbers of women in each group are shown in parentheses.
*$P < 0·05$

of other variables associated with reproduction (Table VI). There appeared to be no association between risk and age at menarché in any of the histological categories. Age at which the first pregnancy ended also did not generally appear to be related to risk. The significant linear trend of risk of carcinoma *in situ* with age at the first pregnancy failed to persist after adjustment for number of sexual partners, with the measure of linear trend decreasing from −2·5 to −0·7 after adjustment. For all the categories of cervical abnormality, however, relative risks were lower for those with a late pregnancy than for those with an early pregnancy. There was no clear relationship between number of pregnancies and risk of cervical abnormalities (Table VI) but nulliparous women were at the lowest risk of severe dysplasia and carcinoma *in situ*.

Several other variables reflecting reproductive or marital status were analysed. These included reported pregnancy outside marriage, pregnancy termination, and divorce. Since these were all highly corre-

TABLE VII.—*Relative risks of cervical abnormalities according to selected reproductive and marital factors*

| | Controls (422) | Normal histology (47) | Mild dysplasia (44) | Severe dysplasia (81) | Carcinoma *in situ* (65) |
|---|---|---|---|---|---|
| Pregnancy outside marriage | | | | | |
| % Positive | 22·7 | 23·4 | 38·6 | 45·7 | 56·9 |
| Relative risk† | 1·00 | 0·50 | 1·27 | 1·47 | 2·58 |
| 95% CI | — | (0·2–1·2) | (0·6–2·7) | (0·9–2·4) | (1·5–4·5) |
| Ever had pregnancy terminated | | | | | |
| % Positive | 6·2 | 12·8 | 22·7 | 8·6 | 10·8 |
| Relative risk† | 1·00 | 1·44 | 2·76 | 0·79 | 1·03 |
| 95% CI | — | (0·5–3·9) | (1·1–6·8) | (0·3–1·9) | (0·4–2·5) |
| Ever divorced | | | | | |
| % Positive | 10·7 | 27·7 | 18·2 | 19·8 | 27·7 |
| Relative risk† | 1·00 | 2·09 | 1·02 | 0·91 | 1·53 |
| 95% CI | — | (0·9–4·6) | (0·5–2·3) | (0·5–1·8) | (0·8–3·0) |

† Adjusted for number of sexual partners (0–1, 2, 3–5, 6+).

TABLE VIII.—*Relative risks† of cervical abnormalities according to social class and tobacco and alcohol consumption*

| | Normal histology (47) | Mild dysplasia (44) | Severe dysplasia (81) | Carcinoma *in situ* (65) |
|---|---|---|---|---|
| Social class‡ | | | | |
| 1 | 1·00 (8) | 1·00 (3) | 1·00 (9) | 1·00 (7) |
| 2 | 0·80 (12) | 1·81 (10) | 1·09 (18) | 0·96 (12) |
| 3 | 0·76 (21) | 2·58 (27) | 1·17 (38) | 1·34 (33) |
| 4 or 5 | 0·52 (6) | 0·70 (4) | 1·00 (15) | 1·15 (13) |
| $\chi^2$ for linear trend | −1·35 | −0·01 | 0·06 | 0·72 |
| Current smoking (cigarettes/day) | | | | |
| None | 1·00 (31) | 1·00 (15) | 1·00 (28) | 1·00 (25) |
| <15 | 1·04 (6) | 3·14 (10) | 2·70 (14) | 1·95 (10) |
| 15–19 | 1·71 (4) | 1·73 (5) | 3·47 (14) | 3·27 (11) |
| 20–24 | 0·77 (3) | 5·02 (11) | 3·08 (14) | 4·27 (15) |
| 25+ | 0·80 (3) | 1·66 (3) | 5·12 (11) | 2·77 (4) |
| $\chi^2_1$ for linear trend | −0·05 | 3·00** | 4·32*** | 3·38*** |
| Alcohol consumption | | | | |
| Never | 1·00 (5) | 1·00 (3) | 1·00 (2) | 1·00 (5) |
| Monthly | 0·73 (18) | 1·18 (12) | 2·62 (22) | 0·83 (20) |
| Weekly | 0·56 (13) | 1·00 (13) | 3·70 (31) | 0·87 (19) |
| Daily | 0·73 (11) | 1·61 (16) | 4·15 (26) | 1·23 (21) |
| $\chi^2_1$ for linear trend | −0·82 | 1·28 | 2·09* | 0·74 |

† Adjusted for age (<30, 30–39, 40+).
‡ One woman with severe dysplasia excluded from analysis due to missing data.
*$P<0·05$; **$P<0·01$; ***$P<0·001$.

lated with number of sexual partners, it was necessary to adjust for this variable in the analysis (Table VII). Women who reported a pregnancy outside marriage, whether premaritally or at some other time outside legal marriage, were at increased risk. This association was statistically significant only for carcinoma *in situ* (relative risk 2·6). Termination of pregnancy was associated with a significant increase in the relative risk (2·8) only for mild dysplasia. Women who reported having been divorced showed no significantly increased risk of any category of cervical abnormality.

The influence of social class and tobacco and alcohol consumption is shown in Table VIII. There was no clear relationship between social class and risk of cervical abnormality. Cigarette smoking, however, was quite strongly associated with risk. Statistically significant ($P < 0{\cdot}01$) linear trends were obtained with the number of cigarettes currently smoked per day for both categories of dysplasia and for carcinoma *in situ*. Women smoking 20 or more cigarettes had 3–4 times the risk of non-smokers. To determine whether these associations were an artefact due to inadequate adjustment for age (which related strongly to the amount smoked) we made additional analyses controlling for age in 5-year groupings. The associations persisted after finer adjustment for age, and also after adjustment for number of sexual partners. Alcohol consumption, on the other hand, did not generally appear to be related to risk of cervical abnormality. The notable exception was severe dysplasia, where a significant linear trend was observed with increasing frequency of consumption. However, the trend did not remain significant after adjustment for number of sexual partners (measure of linear trend reduced from 2·1 to 1·1).

Further analyses considered the influence of different methods of contraception on the risk of cervical abnormality (Table IX). Statistically significant ($P < 0{\cdot}05$) linear relationships were seen for length of use of oral contraceptives and risk of severe dysplasia and carcinoma *in situ*. A positive association was also found among women with normal histology. These associations remained significant after adjustment for number of sexual partners. In contrast to the findings for oral contraceptives, risk of cervical abnormality *decreased* with increasing years of use of barrier methods of contraception (diaphragm or sheath) but was statistically significant only among women with severe dysplasia. This trend was observed when years of use of the sheath and years of use of the cap were analysed separately and also when the data were adjusted for effects of sexual partners. There appeared to be no relationship of years of use of the intrauterine device to risk of cervical abnormality; however, only 4·0% of the women reported its use for 5 or more years.

Since the analyses so far described suggested that risk factors were broadly similar for cervical dysplasia and carcinoma *in situ*, these groups were combined for analysis of the independence of risk factors. This allowed more stable risk estimates to be derived and enabled the possible influence of a larger number of variables to be considered in the multivariate analyses.

TABLE IX.—*Relative risk† of cervical abnormalities according to duration of use of oral contraceptives and barrier methods of contraception*

| Duration of use (years) | Normal histology (47) | Mild dysplasia (44) | Severe dysplasia (81) | Carcinoma *in situ* (65) |
|---|---|---|---|---|
| Oral contraceptives | | | | |
| Never | 1·00 (17) | 1·00 (13) | 1·00 (15) | 1·00 (19) |
| < 5 | 0·78 (11) | 0·92 (17) | 1·68 (27) | 0·68 (19) |
| 5–9 | 1·88 (13) | 1·17 (7) | 3·61 (31) | 1·19 (22) |
| 10+ | 6·52 (6) | 3·03 (7) | 4·04 (8) | 2·73 (5) |
| $\chi^2_1$ for linear trend | 3·09** | 1·63 | 3·75*** | 2·04* |
| Barrier methods‡ | | | | |
| Never | 1·00 (16) | 1·00 (8) | 1·00 (30) | 1·00 (18) |
| < 5 | 1·13 (16) | 1·55 (19) | 0·86 (35) | 1·14 (27) |
| 5–9 | 0·74 (6) | 2·32 (10) | 0·38 (8) | 1·43 (13) |
| 10+ | 0·39 (9) | 0·59 (7) | 0·17 (8) | 0·52 (7) |
| $\chi^2_1$ for linear trend | −2·01* | 0·60 | −2·58** | −1·04 |

† Relative risks adjusted for age (< 30, 30–39, 40+).
‡ Diaphragm or sheath.
*$P < 0{\cdot}05$; **$P < 0{\cdot}01$; ***$P < 0{\cdot}001$.

We included in the multivariate analyses those variables that were found to be associated with cervical dysplasia or carcinoma *in situ* in the single-variable analyses. These variables were age, number of sexual partners, history of pregnancy outside marriage, history of a termination of pregnancy, cigarette smoking, years of use of oral contraceptives and years of use of barrier methods of contraception. Examination of the coefficients associated with these factors revealed that some were not significantly associated with risk of disease after allowing for the effects of other variables, and these were not included in subsequent models. Step-down and step-up regression techniques provided similar results regarding the variables most strongly related to risk. The final model chosen, as most appropriately identifying the major independent risk factors, is presented in Table X.

TABLE X.—*Logistic analysis of risk factors for cervical abnormalities (cervical dysplasia or carcinoma* in situ)

| | $\beta$ (s.e. $\beta$)* | Relative risk | Likelihood ratio test† |
|---|---|---|---|
| No. sexual partners | | | $\chi^2_3 = 55{\cdot}74$ ($P < 0{\cdot}001$) |
| 0–1 | 0·00 (—) | 1·00 | |
| 2 | 0·86 (0·28) | 2·37 | |
| 3–5 | 1·69 (0·26) | 5·41 | |
| 6+ | 1·81 (0·32) | 6·09 | |
| Pregnancy outside marriage | | | $\chi^2_1 = 5{\cdot}66$ ($P = 0{\cdot}017$) |
| No | 0·00 (—) | 1·00 | |
| Yes | 0·51 (0·21) | 1·67 | |
| Current smoking (cigarettes per day) | | | $\chi^2_3 = 15{\cdot}81$ ($P = 0{\cdot}001$) |
| None | 0·00 (—) | 1·00 | |
| < 15 | 0·77 (0·29) | 2·16 | |
| 15–19 | 0·90 (0·32) | 2·45 | |
| 20+ | 0·75 (0·25) | 2·12 | |
| Years of oral contraceptive use | | | $\chi^2_3 = 14{\cdot}09$ ($P = 0{\cdot}003$) |
| None | 0·00 (—) | 1·00 | |
| < 5 | −0·26 (0·25) | 0·77 | |
| 5–9 | 0·56 (0·28) | 1·75 | |
| 10+ | 0·76 (0·40) | 2·13 | |

* $\beta$ is the estimate of the natural logarithm of the relative risk obtained by fitting a logistic model.

† The $\chi^2$ values indicate the significance of the additional contribution of each variable to the fit of the logistic model after the other 3 variables have already been included. Each of the 4 variables makes a statistically significant contribution to the patterns of risk demonstrated in the final model.

The strongest risk factor for cervical abnormalities was multiple sexual partners, with women reporting 6 or more sexual partners having a risk 6·1 times higher than those with only one partner. Other risk factors that emerged from this analysis were cigarette smoking (smokers had twice the risk of non-smokers) and pregnancy outside marriage (relative risk: 1·7). The use of oral contraceptives also remained a risk factor, the risk being highest among women who had used them for 10 years or longer.

### DISCUSSION

Most of the factors linked with invasive cervical cancer in other epidemiological studies were found to be related to carcinoma *in situ* and dysplasia of the cervix in this study, including early age at first intercourse, multiple sexual partners and pregnancy outside marriage. We also found similarities in risk factors for non-invasive and invasive cervical abnormalities. During the course of this study, 27 women with invasive cancer were interviewed who were of a similar age to women with other cervical abnormalities. On the basis of the distributions of age at first intercourse and number of sexual partners for women of different ages among those with non-invasive abnormalities, we predicted that 8·6 of the 27 women with invasive cancer would have had intercourse before 18 years of age and 12·5 would have had 3 or more sexual partners. In fact, the numbers were 7 and 13 respectively.

In addition to finding similarities in risk factors for invasive and non-invasive cervical abnormalities, we also found that risk factors were similar for mild dysplasia, severe dysplasia and carcinoma *in situ*. Our results support the findings of others (Meisels *et al.*, 1977; Wright *et al.*, 1978) and contrast with those of Thomas (1973). Thomas reported that these factors tended to relate only to carcinoma *in situ* or severe dysplasia, suggesting that these lesions have causes similar to those of invasive carcinoma, whereas mild dysplasia is possibly a nonspecific reaction of the cervical epithelium. Our findings indicate the importance of sexual factors, and thus the possibility of some kind of venereal transmission, in the aetiology of both mild and severe dysplasia as well as carcinoma *in situ*.

Methods of classifying dysplastic lesions of the cervix according to their pathology vary (Editorial, 1975) which may explain the difference between our results and those obtained by Thomas. The pathological criteria used by Thomas are not clear, and it is possible that he would have included some of the patients whom we regarded as having dysplasia in the carcinoma *in situ* group. In our study, even the women with "normal histology" showed some associations with established cervical-cancer risk factors, though the association was generally considerably less than for dysplasia or carcinoma *in situ*. These associations indicate that some women having abnormal smears followed by normal biopsy results may actually be in the early phases of epithelial alteration. Alternatively, it is possible that the weak associations represent some bias in the presentation or clinical selection of patients for a cervical smear, or in the inability of the control group to represent the general population. In view of these possibilities, additional analyses were performed, in which the women with normal histology were used as a control group. In these analyses, the associations with number of sexual partners, age at first intercourse and pregnancy outside marriage persisted for severe dysplasia and for carcinoma *in situ*, providing further support for the hypothesis that sexual factors play a major role in the aetiology of these conditions.

In this study, the number of sexual partners had an effect independent of age at first sexual intercourse, whereas the reverse was not true. This contrasts with the findings of several other investigators with respect to cervical cancer (Rotkin & Cameron, 1968; Singer, 1975) though their methods were not directly comparable to ours. It has been suggested that age at first intercourse is an important risk factor because of the increased vulnerability of the cervix at times when metaplasia is likely, such as during adolescence (Coppleson & Reid, 1968). The finding in this study of a stronger relationship with number of sexual partners appears to indicate a venereal means of transmission that is unaffected by times of increased vulnerability. Whether this also applies to invasive cervical cancer requires further investigation.

Social class did not appear to affect the risk of any of the conditions studied. This is in contrast to a number of studies that have shown social class to relate quite strongly to the risk of cervical cancer (Aitken-Swan & Baird, 1966; Stewart *et al.*, 1966). It seems unlikely that this disparity is due to differences in the aetiology of invasive and non-invasive cervical abnormalities, given that we found the other cervical-cancer risk factors to be such strong risk indicators for cervical dysplasia and carcinoma *in situ*. It is perhaps more likely that sexual behaviour has over time become less strongly influenced by social class. This interpretation is supported by the fact that among the control subjects we found no marked differences in the numbers of sexual partners among women in the different social classes. The variation in the characteristics classified by occupation (and hence by the Registrar-General's categories of social class) may also be somewhat less in Oxfordshire than in other parts of Britain.

The finding that use of oral contraceptives was associated with cervical abnormalities is consistent with several other studies (Meisels *et al.*, 1977; Peritz *et al.*, 1977). Although the results of these studies have been questioned (Thomas, 1978) because of inadequate control for possible confounding factors (such as age at first intercourse and number of sexual partners) the association persisted in the present enquiry after adjustment for several risk factors. The possibility of a direct association must therefore be considered. One problem in interpretation is the appropriateness of the group to which pill users are compared. Initially we thought that our findings might have been due to inclusion of diaphragm users in the control group, since use of the diaphragm may protect against cervical abnormalities, including cancer (Boyd & Doll, 1964; Boyce *et al.*, 1977; Wright *et al.*, 1978). However, users of oral contraceptives still had higher risks than women who had not used barrier methods of contraception. The latter group included women who had never used any form of contraception, and they might be unrepresentative. We therefore conducted a further analysis in which users of the pill were compared with users of other non-barrier methods. There was still a high relative risk (2·6) associated with long-term use of the pill, although the numbers in this analysis were small (9 women with cervical abnormalities, 26 control women). The fact that pill associations were significant even among the women with normal histology might indicate a tendency for long-term users of the pill to be referred by their physicians for a cervical smear. This finding made us consider whether the pill associations were due to problems in the appropriateness of the control group. However, the pill associations remained even when women with menstrual disorders, who showed a slightly lower rate of pill use, were excluded from the control series. The difficulties in evaluating pill associations are compounded by the fact that oral contraceptives may cause eversion of the endocervix, making abnormalities easier to detect (Editorial, 1977). In addition, the decision to use oral contraceptives may be made more often by women with pre-existing dysplasia (Stern & Coffelt, 1970; Ory *et al.*, 1976). The question whether the pill is causally related to cervical neoplasia must await further research.

The fact that smoking persisted as a strong risk factor after adjustment for several other risk factors was surprising, because we expected that any association with smoking would be indirect, reflecting the influence of correlated sexual characteristics. Thomas (1973) and Wright *et al.* (1978) also found smoking to remain as a risk factor for carcinoma *in situ* and other cervical neoplasms after adjustment for several established risk factors. Although a causal relationship seems biologically implausible, and we did not find that risk among smokers increased with the number of cigarettes smoked (Table X) the association possibly deserves further investigation. Future studies should evaluate whether smoking associations are influenced by vitamin A (or $\beta$ carotene) intake, suggested since recent research has shown a protective effect of these dietary factors for several tumours of squamous cell type.

In summary, our findings suggest that sexual factors appear to be important in the aetiology of non-invasive cervical abnormalities. Whether lesions such as cervical dysplasia and carcinoma *in situ* are true precursors of invasive cervical carcinoma can be demonstrated only in cohort studies. The similarities in risk factors, however, support this hypothesis. In addition to sexual factors, this study has identified two other factors that may be associated with the risk of cervical abnormalities: use of oral contraceptives and smoking. We were unable to show that these associations were incidental, and they deserve further study.

We are indebted to the consultant gynaecologists at the Churchill and John Radcliffe Hospitals for allowing us to interview their patients, to Dr Keith Hartman for providing additional information about patients undergoing colposcopy, and to Sister Winch and the other nursing staff for their assistance with the notification of patients.

We would like to thank Dr Leo Kinlen for his help. Also, we are grateful to Paul Humphreys, Carol Hermon and Andy Scott for valuable help in computer programming.

Dr Brinton's work for this paper was undertaken during a Research Training Fellowship awarded by the International Agency for Research on Cancer.

### REFERENCES

AITKEN-SWAN, J. & BAIRD, D. (1966) Cancer of the uterine cervix in Aberdeenshire. Aetiological aspects. *Br. J. Cancer*, **20**, 642.

ANDERSON, J. A. (1972) Separate sample logistic discrimination. *Biometrika*, **59**, 19.

BOYCE, J. G., LU, T., NELSON, J. H. & FRUCHTER, R. G. (1977) Oral contraceptives and cervical carcinoma. *Am. J. Obstet. Gynecol.*, **128**, 761.

BOYD, J. T. & DOLL, R. (1964) A study of the aetiology of carcinoma of the cervix uteri. *Br. J. Cancer*, **18**, 419.

COPPLESON, M. & REID, B. (1968) The etiology of squamous carcinoma of the cervix. *Obstet. Gynecol.*, **32**, 432.

EDITORIAL (1975) Cervical epithelial dysplasia. *Br. Med. J.*, i, 294.

EDITORIAL (1977) Cervical neoplasia and the pill. *Lancet*, ii, 644.

FLEISS, J. (1973) *Statistical Methods for Rates and Proportions*. New York: John Wiley.

GOVAN, A. D. T., HAINES, R. M., LANGLEY, F. A., TAYLOR, C. W. & WOODCOCK, A. S. (1969) The histology and cytology of changes in the epithelium of the cervix uteri. *J. Clin. Pathol.*, **22**, 383.

KESSLER, I. I. (1976) Human cervical cancer as a venereal disease. *Cancer Res.*, **36**, 783.

MANTEL, N. (1963) Chi-square tests with one degree of freedom, extensions of the Mantel–Haenszel procedure. *J. Am. Stat. Assn*, **58**, 690.

MANTEL, N. & HAENSZEL, W. (1959) Statistical aspects of the analysis of data from retrospective studies of disease. *J. Natl Cancer Inst.*, **22**, 719.

MEISELS, A., BÉGIN, R. & SCHNEIDER, V. (1977) Dysplasias of the uterine cervix. Epidemiological aspects: Role of age at first coitus and use of oral contraceptives. *Cancer*, **40**, 3076.

MIETTINEN, O. S. (1976) Estimability and estimation in case-referent studies. *Am. J. Epidemiol.*, **103**, 226.

ORY, H., NAIB, Z., CONGER, S. B., HATCHER, R. A. & TYLER, C. W. (1976) Contraceptive choice and prevalence of cervical dysplasia and carcinoma *in situ*. *Am. J. Obstet. Gynecol.*, **124**, 573.

PERITZ, E., RAMCHARAN, S., FRANK, J., BROWN, W. L., HUANG, S. & RAY, R. (1977) The incidence of cervical cancer and duration of oral contraceptive use. *Am. J. Epidemiol.*, **106**, 462.

ROTKIN, I. D. (1967) Adolescent coitus and cervical cancer: Associations of related events with increased risk. *Cancer Res.*, **27**, 603.

ROTKIN, I. D. & CAMERON, J. R. (1968) Clusters of variables influencing risk of cervical cancer. *Cancer*, **21**, 663.

SAMSON, C. D., WAKEFIELD, J. & YULE, R. (1971) Trends in cytological screening in the Manchester area, 1965 to 1971. *Community Med.*, **126**, 253.

SINGER, A. (1975) The uterine cervix from adolescence to the menopause. *Br. J. Obstet. Gynaecol.*, **82**, 81.

STERN, E. & COFFELT, C. F. (1970) Contraceptives and dysplasia: Higher rate for pill choosers. *Science*, **169**, 497.

STEWART, H. L., DUNHAM, L. J., CASPER, J., DORN, H. F., THOMAS, L. B., EDGCOMB, J. H. & SYMEONIDIS, A. (1966) Epidemiology of cancers of uterine cervix and corpus, breast and ovary in Israel and New York City. *J. Natl Cancer Inst.*, **37**, 1.

THOMAS, D. B. (1973) An epidemiologic study of carcinoma *in situ* and squamous dysplasia of the uterine cervix. *Am. J. Epidemiol.*, **98**, 10.

THOMAS, D. B. (1978) Role of exogenous female hormones in altering the risk of benign and malignant neoplasms in humans. *Cancer Res.*, **38**, 3991.

WAKEFIELD, J., YULE, R., SMITH, A. & ADELSTEIN, A. M. (1973) Relation of abnormal cytological smears and carcinoma of the cervix uteri to husband's occupation. *Br. Med. J.*, **2**, 142.

WRIGHT, N. H., VESSEY, M. P., KENWARD, B., MCPHERSON, K. & DOLL, R. (1978) Neoplasia and dysplasia of the cervix uteri and contraception: a possible protective effect of the diaphragm. *Br. J. Cancer*, **38**, 273.

WYNDER, E. L., CORNFIELD, J., SCHROFF, P. D. & DORAISWAMI, K. R. A. (1954) A study of environmental factors in carcinoma of the cervix. *Am. J. Obstet. Gynecol.*, **68**, 1016.